# Joint Clustering and Power Optimization for the SPMA Protocol in UAV Swarm Communication in Frequency-Constrained Scenarios

**DOI:** 10.3390/s26092760

**Published:** 2026-04-29

**Authors:** Yu Wu, Changheun Oh, Hongshan Nie, Byung-Seo Kim

**Affiliations:** 1 School of Engineering Science, Shandong Xiehe University, Jinan 250107, China; wuyu1@sdxiehe.edu.cn (Y.W.); hsnie@hnu.edu.cn (H.N.); 2 Department of Software & Communications Engineering, Hongik University, Sejong City 30016, Republic of Korea; choh@hongik.ac.kr

**Keywords:** UAV swarm, SPMA protocol, frequency-constrained, clustering, power control, joint optimization

## Abstract

Unmanned aerial vehicle (UAV) swarms face significant performance degradation when operating on a single-frequency channel, as the Statistical Priority-based Multiple Access (SPMA) protocol suffers from intensified contention conflicts due to scarce frequency resources. To address this issue, this paper proposes a joint clustering and power optimization method for the SPMA protocol in frequency-constrained scenarios. First, a utility function centered on the end-to-end transmission success rate is constructed, and the optimal clustering scheme selection is formulated as a constrained combinatorial optimization problem. Second, a three-stage heuristic algorithm is designed; all iterations are executed virtually at network initialization. K-means is used to perform initial clustering and determine the minimum power required for intra-cluster services, GPSR is used to establish multi-hop routes for inter-cluster services, and the ant colony algorithm refines the transmission power of forwarding nodes, achieving joint optimization of cluster structure and power configuration. Simulation results show that, compared with the standalone SPMA protocol and the typical clustering algorithm ICW, the proposed algorithm reduces transmission power by 90.4% relative to SPMA (with slightly higher power than ICW) and achieves a comprehensive improvement over both benchmarks. Specifically, the success rate is improved by 63.5% compared with SPMA and 162.3% compared with ICW under high traffic loads, thus achieving a well-balanced compromise between power consumption and transmission reliability. This verifies the feasibility and effectiveness of the proposed optimization method in frequency-constrained scenarios.

## 1. Introduction

UAV swarms have provided new application scenarios and technical solutions due to their advantages of low cost, large-scale deployment capability, high autonomy, and multi-platform collaboration [1,2]. With the continuous increase in the number of UAVs in swarm networks, network density is growing, and the types, scale, and quality requirements of services supported by the network are constantly rising, thereby imposing new and higher demands on the networking communication capabilities of UAV swarms.

The SPMA protocol is an emerging and promising wireless MAC protocol that is widely used in UAV networks [3]. By comprehensively adopting mechanisms such as channel load statistics, multi-priority processing, and random contention access, the SPMA protocol can ensure that the TTNT network accommodates more than 100 nodes while maintaining a transmission delay of no more than 2 ms and a transmission success rate of no less than 99% for high-priority services [4]. Therefore, compared with two classic MAC protocols (CSMA [5] and TDMA [6]), the SPMA protocol is more suitable for the communication needs of UAV swarm networks.

Current research on the SPMA protocol mainly focuses on the optimization of mechanisms such as channel load statistics, transmission threshold setting, and random backoff, while increasingly adopting machine learning and deep learning methods to intelligently adjust multiple protocol parameters. Equations for the slot transmission probability are derived, and a resource reservation minimization strategy among priorities is adopted to set low-priority thresholds [7]. A dynamic threshold adjustment mechanism and an adaptive backoff window mechanism are designed to improve system throughput and packet transmission success rates [8]. A non-preemptive M/M/1/K queuing model is established, and a percentile scoring system combined with a Q-learning algorithm is designed to optimize protocol parameters based on this model [9]. A channel load prediction model based on a bi-directional long short-term memory (BiLSTM) neural network is developed, which significantly improves prediction accuracy compared with conventional weighted average-based prediction methods [10]. An Adaptive Credit-Based Shaper with Reinforcement Learning (ACBS-RL) scheduling mechanism is proposed to allocate more transmission opportunities to low-priority services while preferentially satisfying the quality-of-service requirements of high-priority services, thereby improving overall system throughput [11]. A novel model containing two deep Q-learning networks (DQN) is designed, in which a transmission network is used to increase transmission opportunities for low-priority services and a backoff network is used to optimize the backoff mechanism, achieving better throughput and delay performance [12]. Although the above studies improve the performance of the SPMA protocol to a certain extent, none of them break through the inherent limitation that the SPMA protocol relies on multiple frequency channels for large-scale networking [7,13]. Under the condition of limited frequency resources, existing research is insufficient to meet the demands of large-scale UAV swarm networking in terms of node quantity and traffic volume.

Clustering is an effective approach to optimize network topology, reduce routing overhead, and improve energy efficiency and scalability in wireless ad hoc networks [14,15]. As a typical clustering algorithm, LEACH achieves energy balance through random rotation of cluster heads, but tends to aggravate intra-cluster interference and introduce excessive inter-cluster routing hops in large-scale scenarios [16,17]. HEED improves energy efficiency by introducing residual energy and link quality metrics, but still fails to fully guarantee multi-service QoS in dynamic environments [18]. In recent years, intelligent optimization algorithms have driven clustering techniques toward higher efficiency and adaptability, and mainstream solutions can be divided into two categories. The first category is swarm intelligence-based metaheuristic clustering algorithms, which achieve more stable cluster structures and lower energy consumption via multi-objective optimization: GWO models cluster head election as a wolf pack search problem [19]; MWCRSF implements weighted dynamic cluster head election using a sparrow search algorithm [20]; ICW constructs a centralized clustering framework based on a whale optimization algorithm and exhibits outstanding performance in cluster lifetime and energy consumption optimization [21]. The second category is machine learning clustering algorithms represented by K-means, which are widely used due to their low complexity and fast convergence: a dynamic transmission power adjustment scheme for cluster heads is adopted to enhance link stability [22]; the optimal number of clusters is adaptively determined according to topology changes to realize lightweight and efficient clustering [23].

With the popularization of deep learning (DL) in communication networks, DL-based clustering algorithms have gradually emerged. For example, a DQN-based clustering strategy is proposed for dynamic topology in UAV networks, where deep neural networks are used to replace traditional table lookup methods and effectively solve the curse of dimensionality in large-scale state spaces [24]. A graph neural network (GNN) is combined with the HEED protocol to implement energy-efficient clustering in heterogeneous networks by learning topological relationships [25]. Deep reinforcement learning methods, including Q-learning, DQN, and DDPG, are adopted to achieve cross-layer clustering and cooperative optimization of vertical and horizontal routing in Flying Ad Hoc Networks [26]. Although DL-based clustering has significant advantages in dynamic adaptability, it generally suffers from high computational complexity, weak interpretability, and high deployment cost, making it difficult to be directly applied to UAV swarm networking scenarios with strict resource constraints and high real-time requirements.

Clustering integrated with the SPMA protocol offers three key benefits for large-scale UAV swarm networking. First, clustering reduces inter-cluster data interactions. Periodic broadcast services, such as swarm networking, only communicate within clusters and among a small number of cluster heads, while inter-cluster services exchange data among limited nodes without full-network participation. Second, clustering suppresses mutual interference across clusters. Most member nodes transmit at low power to cover only their cluster heads, resulting in weak or negligible inter-cluster interference and a higher data transmission success rate. Third, clustering boosts network capacity. Reduced inter-cluster interference raises the success rate of simultaneous transmissions under the contention-based SPMA protocol, allowing the network to support more nodes and services. Making full use of these benefits can effectively offset performance degradation caused by frequency constraints and better satisfy communication demands in large-scale UAV networks.

The main contributions and novelty of this paper are as follows:

1. This paper identifies the inherent performance bottleneck of the SPMA protocol in frequency-constrained UAV swarm networks and proposes a unified framework that jointly optimizes clustering structure, routing, and transmission power. To the best of our knowledge, this is the first attempt to systematically address severe channel contention in SPMA-based UAV swarms.
2. This paper constructs a multi-service utility function centered on the end-to-end transmission success rate and formally models the optimal clustering problem as a constrained combinatorial optimization problem. The model quantitatively captures the opposite effects of cluster count on intra-cluster and inter-cluster performance, which provides a solid theoretical basis for subsequent joint optimization.
3. This paper designs a three-stage hybrid heuristic algorithm that integrates clustering, routing refinement, and power adjustment. The proposed pipeline efficiently transforms the original NP-hard problem into a polynomial time solvable form, significantly improving both efficiency and real-time performance.
4. Comprehensive experimental results demonstrate that the proposed scheme achieves significant gains in transmission reliability and power efficiency. The proposed design offers a practical and effective solution for large-scale UAV swarm communication under stringent frequency constraints.

The rest of this paper is organized as follows: Section 2 analyzes the SPMA bottlenecks. Section 3 describes the system model. Section 4 derives the theoretical performance. Section 5 presents the hybrid algorithm. Section 6 shows the simulation results, and Section 7 discusses the comparative advantages. Section 8 concludes the work.

## 2. Overview of SPMA Protocol

**Figure 1:**
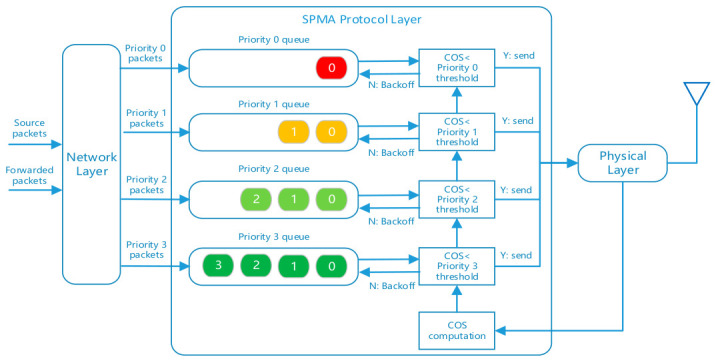
Operational principle of the SPMA protocol.

The operational principle of the SPMA protocol is shown in Figure 1. The network layer places source packets generated by the local node and forwarded packets received from other nodes that require retransmission into the corresponding priority queues of the SPMA protocol based on the priorities of their respective services. The SPMA protocol sequentially checks each priority queue for pending data packets in descending order of priority. If there is a pending packet, it compares the current channel occupancy statistic (COS) with the priority threshold of the queue. If the COS is less than the priority threshold, the packet at the head of the queue is sent to the physical layer for transmission over the channel; otherwise, the queue executes a backoff procedure. When a low-priority queue is in the backoff state, if a higher-priority data packet enters the SPMA protocol, the backoff counting of the low-priority queue needs to be paused, and the transmission judgment for the higher-priority data packet is performed.

**Figure 2:**
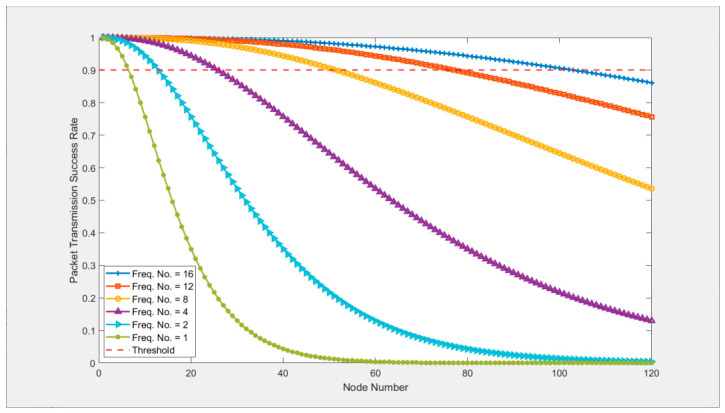
Packet transmission success rate curve of the SPMA protocol.

The priority preemption and channel load statistics mechanisms of the SPMA protocol enable it to meet the low-latency and high-reliability transmission requirements of high-priority services for UAV swarms in multi-frequency scenarios. However, in frequency-constrained single-frequency networking scenarios, channel contention conflicts between nodes are sharply intensified, becoming the core cause of protocol performance degradation. According to the derivation in [7], Figure 2 shows the curve of the SPMA protocol’s packet transmission success rate changing with the number of nodes and frequency channels. It can be seen that when the number of frequency channels is 16, the packet transmission success rate of a UAV swarm network with 100 nodes can still exceed the threshold requirement of 90%; when the number of frequency channels is reduced to 8, the network node scale meeting this threshold is only 51; while in the frequency-constrained scenario of a single-frequency channel (frequency number = 1), the network can only support effective networking of 7 nodes, which is far from meeting the actual application needs of large-scale UAV swarms.

## 3. System Model and Problem Description

### 3.1. System Model

Consider a UAV swarm composed of *N* UAV nodes, supporting *M* types of priority services, with the entire network only supporting one communication frequency channel. The transmission power of UAV nodes can be adjusted in *P* levels within the range $\left[p_{min},p_{max}\right]$. When each node takes off, it first uses the maximum transmission power to form a fully connected network and broadcasts its geographic location information through the highest-priority swarm networking service. The predefined global control node executes the power-controlled clustering algorithm in a centralized manner based on the location information of each node and the priority services to be run by each node, and broadcasts the calculated optimal clustering scheme and power configuration scheme to the entire network through swarm networking service packets, thereby reconstructing the fully connected network into $K\in [K_{min},K_{max}]$ clusters with minimal mutual interference. Denote the clustering set as $\left\{{CS}_{0},{CS}_{1},\ldots ,{CS}_{K-1}\right\}$, where all nodes belong to exactly one cluster, and each cluster ${CS}_{k}$ has exactly one cluster head node and the rest are member nodes. The basic requirements for clustering and power configuration are: member nodes in each cluster can communicate directly with the cluster head; any cluster heads can communicate directly or in multi-hop form; to support specific inter-cluster services, the source and destination nodes of the service can communicate directly or in multi-hop form, and the nodes completing the forwarding can be either cluster head nodes or member nodes.

**Figure 3:**
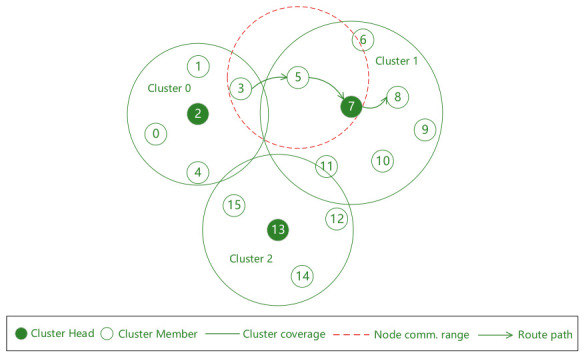
Network topology diagram with three clusters.

The core advantage of forming a clustered network through power control is that a smaller transmission power can be adopted according to the communication needs of specific services, confining interference to a small range, significantly improving the intra-cluster transmission success rate, and also increasing the success rate of simultaneous transmission between different clusters, thereby increasing the network capacity. As shown in Figure 3, when node 0 broadcasts its swarm networking service data to node 2, it will only be interfered with by nodes 1, 3 and 4; moreover, nodes 0 and 10 can broadcast their respective swarm networking service data simultaneously without causing interference to each other’s receiving nodes.

**Figure 4:**
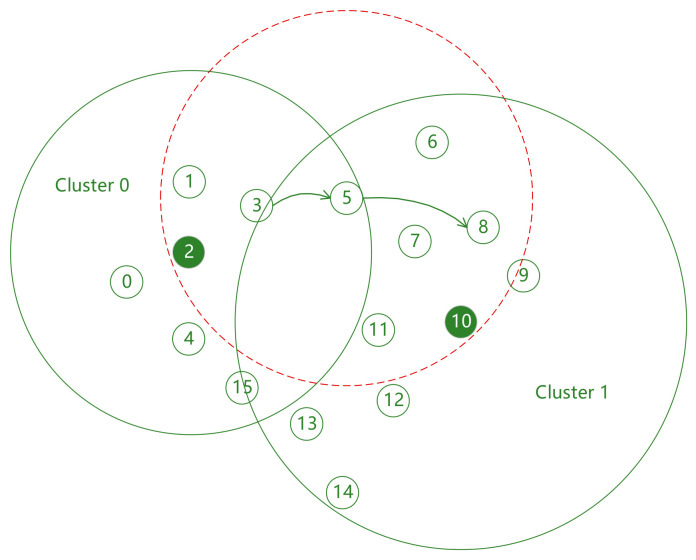
Network topology diagram with two clusters.

However, on the other hand, more clusters are not always better. Since cluster head nodes also need to broadcast swarm networking services, as the number of clusters increases, the number of cluster head nodes also increases, which will inevitably lead to a decrease in the transmission success rate of swarm networking services between cluster heads. At the same time, there are many other types of services in UAV swarms that need to communicate between any two nodes in the entire network. Under power control, multi-hop transmission is usually required. Obviously, the more clusters there are, the smaller the transmission power of member nodes, and the lower the end-to-end transmission success rate. As shown in Figure 3, when node 3 transmits situational awareness services to node 8, three hops are required; while if divided into two clusters as shown in Figure 4, only two hops are needed.

It can be seen that the number of clusters, clustering method, and power configuration have a direct impact on the quality of service of various services. Among them, the number of clusters has a crucial and opposite impact on the quality of service of intra-cluster and inter-cluster services. Therefore, when both types of services exist, the appropriate number of clusters must be a compromise value. To determine the optimal clustering scheme, the following optimization objective is to maximize the quality of service across all services.

It should be clarified that the proposed system uses a centralized decision-making framework relying on global location information and predefined service requirements. It is suitable for UAV swarms with a leader or command node in formation flight and cooperative missions. The global control node only executes clustering and power optimization once at network initialization, so it introduces no communication bottlenecks or extra signaling overhead. This work focuses on verifying the theoretical feasibility and performance gain of joint optimization for the SPMA protocol in frequency-constrained scenarios.

### 3.2. Problem Description

Define ${QoS}_{i,s,K}$ as the quality of service obtained by priority service *s* supported by node *i* under clustering scheme $C_{K}$:

(1) $${QoS}_{i,s,K}=\frac{P_{i,s,K}^{e}}{P_{s}^{req}},$$

where $P_{i,s,K}^{e}$ denotes the end-to-end transmission success rate of priority service *s* data packets sent by node *i* under clustering scheme $C_{K}$, and $P_{s}^{req}$ denotes the required packet transmission success rate of priority service *s*.

On this basis, let the utility function $U_{K}$ represent the weighted sum of the quality of service of all priority services under clustering scheme $C_{K}$:

(2) $$U_{K}=\sum_{s=0}^{M-1}\alpha_{s}\cdot \bar{{QoS}_{i,s,K}},i\in \left\{0,1,\ldots ,N-1\right\},$$

where $\bar{{QoS}_{i,s,K}}$ denotes the average value of ${QoS}_{i,s,K}$ of all nodes supporting priority service *s*, $\alpha_{s}$ denotes the weight of priority *s*, and $\sum_{s=0}^{M-1}\alpha_{s}=1$. Then, the problem of selecting the optimal clustering scheme is modeled as choosing the clustering scheme with the maximum utility function under several constraints:

(3) $$C_{K^{*}}=arg\;\underset{C_{K}}{\max}U_{K},$$

(4) $$s.t.\;\sum_{k=1}^{K}z_{i,k}=1,\forall i\in \left\{0,1,\ldots ,N-1\right\},$$

(5) $$\sum_{i=0}^{N-1}{z_{i,k}\cdot c}_{i,k}=1,\forall {CS}_{k}\in \{{CS}_{0},{CS}_{1},\ldots ,{CS}_{K-1}\},$$

(6) $$p_{min}\leq p_{i}\leq p_{max},\forall i\in \left\{0,1,\ldots ,N-1\right\},$$

where $z_{i,k}\in \left\{0,1\right\}$ indicates whether node *i* belongs to the *k*-th cluster ${CS}_{k}$, and $c_{ik}\in \left\{0,1\right\}$ indicates whether node *i* is the cluster head node of ${CS}_{k}$. Therefore, Equation (4) ensures that each node belongs to exactly one cluster, Equation (5) ensures that each cluster has exactly one cluster head, and Equation (6) ensures that the transmission power $p_{i}$ of any node *i* is within the range $\left[p_{min},p_{max}\right]$.

In the following section, theoretical modeling and quantitative analysis are performed on the end-to-end packet transmission success rate of the SPMA protocol and the optimal clustering scheme in clustered multi-hop networks.

## 4. Theoretical Analysis of the Optimal Clustering Scheme

### 4.1. Queuing Model of the SPMA Protocol

**Figure 5:**
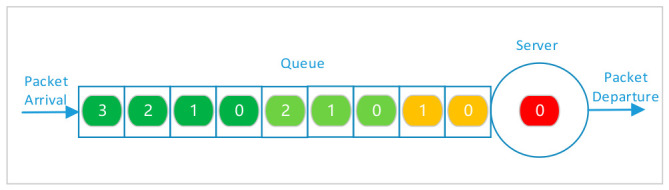
M/G/1 queuing model of the SPMA protocol.

The operational logic of the SPMA protocol in clustered UAV swarm networks can be modeled by a preemptive M/G/1 queuing model, as illustrated in Figure 5. In this tailored model, the notation “M” denotes that the arrival process of data packets (both source packets and forwarded packets) adheres to an independent Poisson distribution, consistent with the randomness of UAV mission traffic. “G” represents that the service time of data packets follows a general distribution, considering the dynamic variations in transmission latency caused by cluster topology changes and power control adjustments. The symbol “1” indicates that each node operates with a single-frequency channel, corresponding to a single-server configuration in the queuing model—meaning the departure process of data packets follows a single-channel transmission constraint, where only one data packet can be transmitted at any given time. Consistent with the preemptive priority design of the SPMA protocol, the queuing model enforces a strict scheduling rule: data packets with higher service priorities are granted absolute transmission precedence and are placed at the head of the queue. For packets of the same priority, the first-come-first-served (FCFS) principle is adopted to ensure fair access to the channel, where packets arriving earlier are scheduled for transmission prior to subsequent ones.

In the following section, the M/G/1 queuing model is used to derive the end-to-end transmission success rate of data packets in the SPMA protocol in multi-hop and clustered networks.

### 4.2. Slot Transmission Probability

Assume that the arrival process of data packets of each priority service generated by each node in the network as a source node follows an independent Poisson distribution. Then, the probability that node *i* generates *k* data packets of priority *s* within time *t* is:

(7) $$q_{i,s}\left(k,t\right)=\frac{e^{-\lambda_{s}\cdot t}\cdot {\left(\lambda_{s}\cdot t\right)}^{k}}{k!},$$

where $\lambda_{s}$ is the arrival rate of data packets of the *s*-th type of service.

In the clustered network topology, the data packets entering the priority transmission queue of node *i* include not only those generated by node *i* itself but also those generated by other nodes that need to be forwarded by node *i*. Therefore, define $\Psi_{i,s}$ as the set of all source nodes that generate priority *s* services and require forwarding by node *i*. Then, the arrival rate of data packets of the *s*-th type of service that need to be forwarded by node *i* is $|\Psi_{i,s}|\cdot \lambda_{s}$. As a result, the total probability that *k* data packets of priority *s* enter node *i* within time *t* is:

(8) $$q_{i,s}^{\prime }\left(k,t\right)=\frac{e^{-\left(\left|\Psi_{i,s}\right|+1\right)\cdot \lambda_{s}\cdot t}\cdot {\left(\left(\left|\Psi_{i,s}\right|+1\right){\cdot \lambda }_{s}\cdot t\right)}^{k}}{k!}.$$

Given the preemptive scheduling nature of the SPMA protocol, low-priority data packets in a backoff state face a key constraint: once a higher-priority data packet is admitted into the transmission queue, the low-priority packet must immediately pause its backoff counting process. This pause mechanism ensures that higher-priority service gains unimpeded transmission priority—an essential design to meet the low-latency demands of critical services such as swarm networking in clustered UAV communication scenarios. For data packets of service type *s*, the likelihood that no higher-priority service arrives within a time window *t* is expressed as:

(9) $$f_{i,s}\left(t\right)=\left\{\begin{array}{c} \prod_{m=0}^{s-1}q_{i,m}^{\prime }\left(0,t\right)=\prod_{m=0}^{s-1}e^{-(|\Psi_{i,m}|+1)\cdot \lambda_{m}\cdot t},\;\;\;\;s\geq 1\\ 1,\;\;\;\;\;s=0. \end{array}\right.$$

Based on the above equation, we can obtain the total number of slots in the backoff phase before the data packet of priority *s* at node *i* is transmitted without being preempted:

(10) $$\bar{X_{i,s}}=\left\{\begin{array}{c} \sum_{j=1}^{B}l_{j}{\left(\gamma_{i,s}\right)}^{j}\prod_{a=1}^{j}f_{i,s}\left(l_{a}\right)=\sum_{j=1}^{B}l_{j}{\left(\gamma_{i,s}\right)}^{j}\prod_{a=1}^{j}\prod_{m=0}^{s-1}e^{-(|\Psi_{i,m}|+1){\cdot \lambda }_{m}\cdot l_{a}},\;\;\;\;s\geq 1\\ \sum_{j=1}^{B}l_{j}{\left(\gamma_{i,s}\right)}^{j},\;\;\;\;\;s=0, \end{array}\right.$$

where $\gamma_{i,s}$ denotes the backoff probability of data packets of priority *s* at node *i*; *B* is the maximum number of backoffs; $l_{a}$ is the length of the *a*-th backoff window, in slots. $\prod_{a=1}^{j}f_{i,s}\left(l_{a}\right)$ denotes the probability that no higher-priority data packets arrive from the 1st to *j*-th backoff process. For data packets of priority 0, since there is no higher priority, this product term is always 1.

In the above equation, the first line on the right-hand side represents the total number of slots in the backoff phase before non-highest-priority data packets are transmitted without being preempted. The second line represents the total number of slots in the backoff phase before the highest-priority data packets are transmitted. For the highest-priority data packets, since there are no higher-priority data packets in the system, their backoff process will not be affected by the arrival of data packets. However, if the channel load is higher than the priority threshold, it will enter backoff.

The average probability that a packet of priority *s* is transmitted after *j* backoffs is:

(11) $$E_{i,s,j}=\left\{\begin{array}{c} 1-\gamma_{i,s},\;\;\;\;j=0\\ {{(\gamma }_{i,s})}^{j}\prod_{a=1}^{j}f_{i,s}\left(l_{a}\right)(1-\gamma_{i,s}),\;\;\;\;\;1\leq j\leq B. \end{array}\right.$$

In the above equation, the first line represents the probability that a data packet of priority *s* is transmitted directly without backoff because the channel load is lower than its priority threshold; the second line represents the probability that a data packet of priority *s* is transmitted after 1 to *B* backoffs. Based on the above equation, the probability that the data packet is transmitted before the arrival of a higher-priority packet is:

(12) $$E_{i,s}=\sum_{j=0}^{B}E_{i,s,j}.$$

Since the arrival process of data packets of priority *s* entering node *i* follows a Poisson distribution with an arrival rate of $(\left|\Psi_{i,s}\right|+1)\cdot \lambda_{s}$, according to the characteristics of the M/G/1 queue, the traffic intensity of data packets of priority *s* is $\eta_{i,s}$, where $\eta_{i,s}\;\;(\left|\Psi_{i,s}\right|+1){\cdot \lambda }_{s}\;\cdot \bar{X_{i,s}}$. The total traffic intensity of all priorities is:

(13) $$\eta_{i}=\sum_{s=0}^{M-1}\eta_{i,s}.$$

The probability $\phi_{i,s}$ that a data packet of priority *s* is at the head of the waiting queue of node *i* is:

(14) $$\phi_{i,s}=\frac{\eta_{i,s}\;}{\eta_{i}}.$$

Combining the above equations, it can be found that the probability that node *i* has a data packet transmitted in any slot, i.e., the slot transmission probability $P_{i}$ of node *i*, is a function closely related to the backoff probability $\gamma_{i,s}$. Its expression is:

(15) $$P_{i}\left(\gamma_{i,s}\right)=\sum_{s=0}^{M-1}\phi_{i,s}\cdot E_{i,s}\cdot \frac{1}{\bar{X_{i,s}}}.$$

From a physical perspective, the slot transmission probability intuitively reflects the possibility that a node successfully transmits data packets within a unit slot and is also a core indicator for measuring the intensity of channel contention: when this probability is high, it indicates that the node has a higher probability of successfully seizing resources in channel contention, which indirectly reflects that the channel load is relatively low; on the contrary, the lower the slot transmission probability, the more intense the channel contention, and the higher the probability that the node fails to transmit due to the triggering of the backoff mechanism and data packet collision.

Based on the core role of this indicator, the following will specifically derive the calculation formula of the backoff probability of data packets of different priorities and further analyze its quantitative correlation with the slot transmission probability.

### 4.3. Priority Backoff Probability

Define the neighbor set of node *i* in the clustered network topology as $\Phi_{i}$ (including node *i* itself). If a data packet arrives at the head of the waiting queue of node *i*, the node first counts the number of data packets present on the channel within the occupancy statistics window $U_{s}$. This count is denoted as $X_{p}$ and serves as the COS, with $U_{s}$ measured in slots. Then, compare $X_{p}$ with the priority threshold of the data packet. If $X_{p}$ is greater than the priority threshold, the data packet enters the backoff state; if $X_{p}$ is less than the priority threshold, the data packet can be transmitted. Since the nodes are mutually independent, $X_{p}$ can be considered to follow a binomial distribution $B(n,\bar{P_{i,out}})$, where $\bar{P_{i,out}}$ is the average value of the probability that all nodes in $\Phi_{i}$ transmit data packets in any slot:

(16) $$\bar{P_{i,out}}=\frac{\sum_{j\in \Phi_{i}}P_{j,out}}{{|\Phi }_{i}|},$$

where $P_{j,out}$ is the probability that node *j* transmits data packets in any slot, and *n* is the total number of slots:

(17) $$n=\left|\Phi_{i}\right|{\cdot U}_{s}.$$

Then, the probability that $X_{p}=k$ is:

(18) $$P\left(X_{p}=k\right)=C_{n}^{k}\cdot {\left(\bar{P_{i,out}}\right)}^{k}\cdot {\left(1-\bar{P_{i,out}}\right)}^{n-k}.$$

Let $D_{s}$ be the priority threshold of priority *s*. When $D_{s}$ is less than $X_{p}$, the data packet of priority *s* will enter the backoff state. Therefore, the expression of the backoff probability with respect to $\bar{P_{i,out}}$ can be obtained:

(19) $$\gamma_{i,s}\left(\bar{P_{i,out}}\right)=1-\sum_{k=0}^{D_{s}}P\left(X_{p}=k\right),\;\;\;0\leq s\leq M-1.$$

The above equation indicates that for a given slot transmission probability $\bar{P_{i,out}}$, the backoff probability $\gamma_{i,s}$ for priority *s* at node *i* can be calculated. Substituting $\gamma_{i,s}$ into Equation (15) yields a new slot transmission probability $P_{i}(\gamma_{i,s})$. Its physical implication is as follows: when the initial slot transmission probability of each node is relatively low, the channel load is low, leading to a small backoff probability for each priority. In this case, more data packets of services with all priorities can be transmitted, which allows the slot transmission probability to increase further. However, as the slot transmission probability rises, the channel load increases accordingly, resulting in a larger backoff probability for each priority. When the backoff probability increases to a certain extent, the slot transmission probability will cease to rise, and at this point, the slot transmission probability of each node will reach a balanced value, namely ${P_{i,out}=P}_{i}\left(\gamma_{i,s}\right)$:

(20) $${P_{i,out}=P}_{i}\left(\gamma_{i,s}\right)=P_{i}\left(\gamma_{i,s}\left(\bar{P_{i,out}}\right)\right).$$

The balanced value given by the above equation represents the maximum achievable slot transmission probability under the given network scale and service requirements of the UAV swarm.

### 4.4. End-to-End Packet Transmission Success Rate

Factors affecting the end-to-end packet transmission success rate include the number of hops and the collision probability of each hop. As shown in Figure 3, the end-to-end transmission success rate of inter-cluster service data packets sent from node 3 to node 8 should be the product of the transmission success rates from node 3 to node 5, node 5 to node 7, and node 7 to node 8. Moreover, the factors affecting the single-hop transmission success rate are all transmitting nodes that cause interference to the receiving node of this hop, i.e., the neighbor set of this receiving node.

First, calculate the packet transmission success rate in the single-hop case.

The SPMA system adopts a packet-splitting time-hopping and frequency-hopping mechanism. In this case, assume that each packet has the same length. After channel coding at the physical layer, it is split into $N_{b}$ data frames, which are randomly transmitted on $N_{f}$ frequency channels. The duration of each data frame is one slot, and the slot length is $T_{s}$. For any receiving node *i*, let node *j* be a node in its neighbor set $\Phi_{i}$. Since we have derived that the probability that node *j* has a data packet transmitted in any slot is $P_{j,out}$, node *j* can send $P_{j,out}/T_{s}$ data packets per unit time. Furthermore, the number of data frames sent by all nodes in $\Phi_{i}$ on a single channel per unit time is:

(21) $$\lambda_{i}^{\prime }=\frac{N_{b}\cdot \sum_{j\in \Phi_{i}}P_{j,out}}{T_{s}\cdot N_{f}}.$$

Assuming that the arrival interval of data frames on each channel still follows a Poisson distribution, $\lambda_{i}^{\prime }$ is the arrival rate of the Poisson distribution. Furthermore, since data frames are transmitted in a time-hopping manner, the start times of all data frames over the channel are synchronized. In this case, the transmission success rate of node *i* successfully receiving a data frame is the probability that the number of data frames sent from $\Phi_{i}$ within the $T_{s}$ time when node *i* receives a data frame is 0:

(22) $$P_{i,bs}=e^{-\lambda_{i}^{\prime }\cdot T_{s}}.$$

To correctly decode a complete data packet, node *i* needs to successfully receive at least half of the split data frames. Therefore, the packet transmission success rate of node *i* successfully receiving a data packet is:

(23) $$P_{i,s}=\sum_{n=N_{b}/2}^{N_{b}}C_{N_{b}}^{n}{\cdot {\left(P_{i,bs}\right)}^{n}(1-P_{i,bs})}^{N_{b}-n}.$$

Observing Equations (21)–(23), it can be found that the packet transmission success rate is proportional to the number of frequency channels and inversely proportional to the number of nodes in the neighbor set and the slot transmission probability of each node. That is, the fewer the number of frequency channels, the higher the collision probability on a single channel. However, forming a clustered structure through power control can reduce the number of neighboring nodes for each node, thereby compensating for the loss caused by the insufficient number of frequency channels. Moreover, the more clusters there are, the fewer the number of neighboring nodes for each node, the lower the collision probability, and the higher the transmission success rate.

Based on the single-hop packet transmission success rate, assume that the destination node of the priority service *s* sent by the source node $n_{0}$ is $n_{h}$, and the end-to-end multi-hop route under the clustering scheme $C_{K}$ is {$n_{0}$, $n_{1}$, …, $n_{h}$}. Then, the end-to-end packet transmission success rate of this multi-hop data packet is:

(24) $$P_{n_{0},s,K}^{e}=\prod_{j=1}^{h}P_{n_{j},s}.$$

From the above equation, it can be found that the more hops there are, the lower the transmission success rate of inter-cluster service data packets. Moreover, during the forwarding of data packets by intermediate nodes, interference will also be generated for the neighboring nodes of the intermediate nodes. Therefore, it is necessary to reduce the number of forwarding hops for inter-cluster services, that is, reduce the number of clusters. Overall, Equations (23) and (24) put forward completely opposite requirements on the number of clusters. Therefore, to balance the packet transmission success rates of intra-cluster and inter-cluster services, a compromise number of clusters must be selected.

### 4.5. Complexity of the Optimal Solution

Although the optimal clustering scheme can theoretically be obtained through exhaustive search, in a network with *N* nodes, the number of clustering schemes is given by the Bell number $B_{N}$, where $B_{N}$ denotes the *N*-th Bell number; further, since each node has *P* optional transmission power levels, the total number of clustering schemes combined with power control is as many as $B_{N}{\cdot P}^{N}$. Since $B_{N}$ grows super-exponentially and $P^{N}$ grows exponentially, the computational complexity of this optimization problem is extremely high, indicating that it belongs to an NP-hard problem. Therefore, exhaustive search is not feasible in engineering, and only heuristic algorithms can be used to find suboptimal solutions within polynomial time.

## 5. Clustering and Power Control Algorithm Based on K-Means and Ant Colony Optimization

### 5.1. Basic Method

To enable the actual UAV swarm system to quickly converge to a high-quality scheme, this paper designs a virtual iterative heuristic algorithm that performs all computations virtually after swarm initialization using only node positions and predefined service requirements, without additional real-time message exchange during iteration; only the final suboptimal clustering and power configuration are broadcast once to the network. The algorithm includes three stages: K-means-based clustering, GPSR (Greedy Perimeter Stateless Routing)-based inter-cluster route establishment, and an ant colony algorithm-based power adjustment.

The algorithm starts with the initial number of clusters *K* = $K_{min}$ and is executed in a centralized manner by the predefined global control node according to the following steps:

Step 1: Use the K-means algorithm to divide the network into *K* clusters and determine the nominal transmission power $p_{i,K}^{nominal}$ of any node *i* according to the requirements of intra-cluster services. The nominal transmission power is not actually assigned to the node.

Step 2: Calculate the route of each inter-cluster service according to the GPSR algorithm. In this process, the nominal transmission power of the nodes participating in transmission and forwarding on the route may be appropriately increased. After the route calculation is completed, the transmitting and forwarding nodes of all routes form an inter-cluster route node set $\Omega_{K}$. At the same time, the nominal transmission power of any node *i* is denoted as $p_{i,K,min}^{nominal}$, which indicates that this power is the minimum power required to meet all intra-cluster and inter-cluster service requirements under the current clustering scheme $C_{K}$.

Step 3: Adopt the ant colony algorithm, using the utility function $U_{K}$ defined in Equation (2) as the fitness $F_{K}$ of the ant colony algorithm, and iteratively adjust the nominal transmission power of each node in the set $\Omega_{K}$. The adjustment interval is $\left[p_{i,K,min}^{nominal},\;p_{max}\right]$. After the iteration is completed, the best fitness obtained is denoted as $F_{K,best}$.

Step 4: Increase the number of clusters *K* and re-execute Step 1 until the maximum number of clusters $K_{max}$ is reached. Calculate the maximum value of the best fitness among all clustering schemes, denoted as $F_{K^{\prime },best}$, and the corresponding $C_{K^{\prime }}$ is the suboptimal clustering scheme obtained by the heuristic algorithm. Based on this, the global control node broadcasts the clustering scheme $C_{K^{\prime }}$ and the corresponding nominal transmission power of each node to the entire network through swarm networking service data packets and finally completes the clustering and the actual power configuration of each node.

The proposed three-stage framework follows a logical and sequential design. K-means clustering first generates the network topology and determines the basic transmission power to guarantee intra-cluster connectivity, thereby establishing a constrained solution space for subsequent procedures. GPSR routing then constructs feasible inter-cluster routes and refines the optimization scope. Finally, the ant colony algorithm performs refined power adjustment to enhance overall system performance and ensure stable convergence. Given that the joint optimization problem is NP-hard, the proposed hierarchical approach efficiently yields high-quality suboptimal solutions with controllable approximation performance. Each stage provides necessary inputs for the next, making the entire framework systematic and well-founded.

### 5.2. K-Means-Based Clustering

Since the purpose of clustering is to reduce mutual interference between different clusters by reducing transmission power, this paper adopts the K-means algorithm based on Euclidean distance as the clustering principle and then completes the preliminary calculation of the nominal transmission power. The specific steps are as follows:

Step 1: Randomly select *K* nodes from the network as initial cluster heads according to the current number of clusters *K*.

Step 2: Calculate the Euclidean distances between other nodes and each cluster head node, and assign each node to the cluster with the closest distance.

Step 3: Calculate the geometric center of all nodes in each cluster, and select the node in the cluster closest to the geometric center as the updated cluster head.

Step 4: If the displacement of the cluster head is less than the iterative convergence threshold ε or the maximum number of iterations *I_K_* is reached, complete the clustering and execute the following steps; otherwise, re-execute Step 2.

Step 5: For the cluster head node $c_{k}$ of any cluster ${CS}_{k}$, the nominal transmission power is calculated based on the free-space propagation model as follows:

(25) $$p_{c_{k},K}^{nominal}=\frac{P_{rx,min\cdot }{\left(4\pi d_{k,max}\right)}^{2}\cdot L}{G_{t}G_{r}\lambda^{2}},$$

where $P_{rx,min}$ is the receiver sensitivity, $d_{k,max}={max}_{i\in {CS}_{k},\;i\neq c_{k}}d(i,c_{k})$ is the maximum distance between $c_{k}$ and other member nodes in the cluster, *L* is the system loss coefficient, $G_{t}$ and $G_{r}$ are the transmit antenna gain and receive antenna gain, respectively, and λ is the electromagnetic wave wavelength.

For any member node *i* of any cluster ${CS}_{k}$, its nominal transmission power is:

(26) $$p_{i,K}^{nominal}=\frac{P_{rx,min\cdot }{\left(4\pi d(i,c_{k})\right)}^{2}\cdot L}{G_{t}G_{r}\lambda^{2}},\forall i\in {CS}_{k},i\neq c_{k}.$$

**Figure 6:**
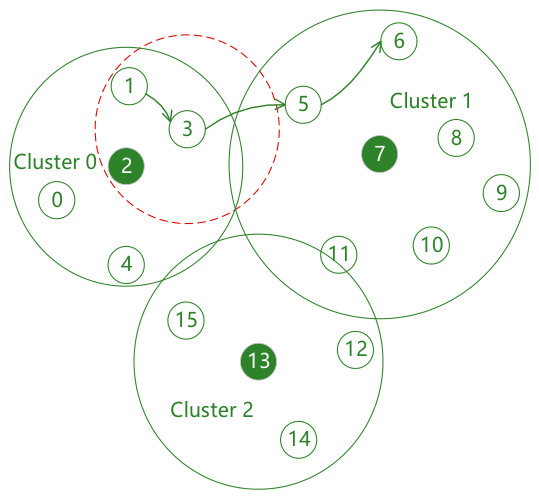
Schematic diagram of K-means-based clustering and power calculation.

After the above steps, as shown in Cluster 0 in Figure 6, the nominal transmission power of the cluster head node 2 can cover all nodes in the cluster, while the nominal transmission power of node 3 as a member is calculated only based on the principle of covering the cluster head.

### 5.3. GPSR-Based Inter-Cluster Route Establishment

The initial calculation of K-means-based clustering and nominal transmission power can only meet the requirements of intra-cluster services. To meet the requirements of inter-cluster services, this paper adopts the GPSR algorithm to establish a route from the source node *src* to the destination node *dst* for each inter-cluster service and appropriately increases the nominal transmission power of the relevant transmitting nodes during the route establishment process.

The algorithm executes the following steps for each inter-cluster service, starting from the source node *src* as the current node *curr*:

Step 1: Calculate the distance $d_{curr\to dst}$ from the current node to the destination node as a reference value;

Step 2: Under the current nominal transmission power of the current node, traverse all its neighboring nodes. If the neighboring nodes include the destination node *dst*, complete the route establishment; otherwise, calculate the Euclidean distance $d_{neigh\to dst}$ from each neighboring node to the destination node;

Step 3: Add all neighboring nodes satisfying $d_{neigh\to dst}<d_{curr\to dst}$ to the candidate next-hop node set of the current node;

Step 4: If the candidate next-hop node set is empty, increase the nominal transmission power $p_{curr,K}^{nominal}$ of the current node by a small step ${∆}_{p}$ and then re-execute Step 2; otherwise, select the neighboring node with the smallest $d_{neigh\to dst}$ as the next-hop node of the current node, add it to the global inter-cluster route node set $\Omega_{K}$, and take this node as the current node *curr*, then re-execute Step 1.

As shown in Figure 6, according to the GPSR algorithm, node 3 is the next-hop node of node 1, where node 1 acts as the source node and node 6 is the destination node. According to the initial nominal transmission power of node 3, its candidate next-hop node set is empty. Therefore, it is necessary to incrementally increase its nominal transmission power until it covers node 5.

### 5.4. Ant Colony Algorithm-Based Power Adjustment

**Figure 7:**
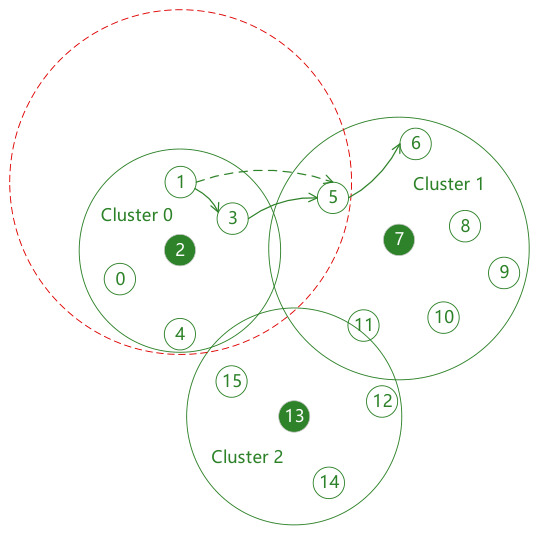
Schematic diagram of power adjustment based on ant colony optimization.

After the establishment of inter-cluster routes, although each inter-cluster service has a route with small transmission power, the number of hops of this route is large, which cannot provide good quality of service. In Figure 6, the number of hops of the route from node 1 to node 6 is 3. However, if the transmission power of node 1 is increased to directly cover node 5 as shown in Figure 7, the number of hops of the route is reduced to 2, thereby significantly improving the end-to-end transmission success rate of this route. However, on the other hand, increasing the transmission power of node 1 will cause more nodes to be interfered with, leading to a decrease in the quality of service of intra-cluster and inter-cluster services related to these nodes. This indicates that the transmission power of relevant nodes cannot be blindly increased only for a certain service. A balanced solution to this problem must be sought from the perspective of the entire network. For this reason, this paper adopts the ant colony algorithm for heuristic solution.

Let each ant in the ant colony algorithm represent a nominal transmission power vector $P_{K}^{nominal}=\left(p_{1,K}^{nominal},\;p_{2,K}^{nominal},\;\ldots ,\;p_{|\Omega_{K}|,K}^{nominal}\right)$, which represents a combination of nominal transmission power values of each node in the set $\Omega_{K}$. Let ${ANT}_{num}$ represent the number of ants used in the ant colony algorithm. Then, in each iteration of the algorithm, ${ANT}_{num}$ ants are released, which is equivalent to generating ${ANT}_{num}$ groups of different nominal transmission power candidate schemes simultaneously to search for the optimal solution in parallel. It should be emphasized that the nominal transmission power of each node in the set $\Omega_{K}$ only takes discrete values based on the route within the range $\left[p_{i,K,min}^{nominal},\;p_{max}\right]$. For example, the nominal transmission power of node 3 in Figure 7 has at most two values within the range not exceeding $p_{max}$, which exactly covers node 5 and node 6, respectively. This is because other values not only do not help improve the quality of service of the inter-cluster service from node 1 to node 6 but also increase interference to other nodes, and this discrete value method can significantly improve the convergence speed of the ant colony search.

Define the fitness function $F_{K}$ of the ant colony algorithm as equal to the utility function:

(27) $$F_{K}=U_{K}\left(P_{K}^{nominal}\right),$$

where $U_{K}\left(P_{K}^{nominal}\right)$ denotes the utility function value determined by the nominal transmission power vector $P_{K}^{nominal}$ during the ant colony iteration.

Let the pheromone matrix of the ant colony algorithm be τ, whose dimension is $\left|\Omega_{K}\right|$ rows × $\underset{i\in \Omega_{K}}{\max}\left(m_{i}\right)$ columns, where $m_{i}$ is the number of discrete values of the nominal transmission power of any node *i*. Initially, all pheromones are set to a constant $\tau_{0}$. After each iteration, the pheromones are updated according to the following rules:

(28) $$\tau_{i,j}\left(t+1\right)=\left(1-\rho \right)\cdot \tau_{i,j}\left(t\right)+\sum_{n=1}^{{ANT}_{num}}∆\tau_{i,j}^{n},$$

where ρ is the pheromone evaporation factor satisfying 0<ρ<1, a larger value of ρ indicates a weaker influence of historical pheromones and thus stronger global search capability; $\tau_{i,j}(t)$ is the pheromone concentration of the *j*-th power of node *i* in the *t*-th iteration; $∆\tau_{i,j}^{n}$ is the pheromone left by the *n*-th ant in this iteration, which is proportional to the quality of the solution found by the ant:

(29) $$∆\tau_{i,j}^{n}=\left\{\begin{array}{c} Q\cdot U_{K}\left(P_{K}^{nominal}\left(n\right)\right),\;\;\;\;\;\;\mathrm{if}\;\mathrm{ant}\;n\;\mathrm{selects}\;\mathrm{power}\;j\;\mathrm{at}\;\mathrm{node}\;i\;\\ 0,\;\;\;\;\;\mathrm{otherwise}, \end{array}\right.$$

where *Q* is the pheromone enhancement coefficient, which is used to adjust the magnitude of the pheromone increment.

Ants select discrete power by combining pheromone guidance and random exploration. For node *i*, the selection probability of its *j*-th discrete power is:

(30) $$P_{i,j}\left(t\right)=\frac{{\left(\tau_{i,j}\left(t\right)\right)}^{\lambda }}{\sum_{l-1}^{m_{i}}{\left(\tau_{i,l}\left(t\right)\right)}^{\lambda }},$$

where λ is the heuristic factor, a larger λ leads to a stronger guiding effect of pheromones and faster convergence, while a smaller λ results in stronger random exploration.

On this basis, the main steps of the ant colony-based power adjustment algorithm are as follows:

Step 1: Release ${ANT}_{num}$ ants in each iteration *t*, and each ant independently completes the following operations:

Step 2: For each node $i\in \Omega_{K}$, calculate the selection probability $P_{i,j}(t)$ of each discrete power *j*;

Step 3: Select the nominal transmission power $p_{i,K}^{nominal}(t)$ of each node using the roulette wheel method to form a set of nominal transmission power vectors $P_{K}^{nominal}(t)$;

Step 4: Calculate the fitness $F_{K}(t,ant)$ corresponding to this vector;

Step 5: Calculate the maximum fitness among all ants in this iteration, denoted as $F_{K,best}(t)$. The vector corresponding to this value, $P_{K,best}^{nominal}(t)$, is the optimal power vector of this iteration. Then, update the pheromone matrix according to the solutions of all ants in this iteration;

Step 6: Increase the number of iterations and re-execute Step 1 until the maximum number of iterations *I_A_* is reached. Calculate the global optimal fitness $F_{K^{\prime },best}$ and obtain the global optimal power vector $P_{K^{\prime },best}^{nominal}$.

### 5.5. Algorithm Complexity Analysis

This section quantitatively analyzes the computational complexity of the proposed heuristic algorithm from three stages: K-means clustering, GPSR inter-cluster route establishment, and ant colony algorithm-based power optimization. The core parameters involved in the analysis include the total number of UAV swarm nodes *N*, the number of clusters *K*, the number of single-round iterations of the K-means algorithm *I_K_*, the total number of service types *M*, the maximum number of hops of GPSR routes *H*, the number of ants in the ant colony algorithm ${ANT}_{num}$, the maximum number of iterations *I_A_*, as well as the scale of the set $\left|\Omega_{K}\right|$ and the discrete value of node power $m_{i}$.

#### 5.5.1. Computational Complexity of the K-Means Clustering Stage

The proposed algorithm traverses the number of clusters $K\in [K_{min},K_{max}]$ and executes the K-means clustering once for each *K*. The computational complexity of a single round of iteration of the classic K-means algorithm is O(N·K), and the main overhead lies in calculating the Euclidean distance between *N* nodes and *K* cluster heads and updating node cluster membership. Then, the computational complexity of K-means clustering under a single *K* value is $O(I_{K}\cdot N\cdot K)$.

When traversing all *K* values, the total computational complexity of the clustering stage is the sum of the complexities corresponding to each *K* value, and the expression is $O\left(I_{K}\cdot N\cdot \sum_{K=K_{min}}^{K_{max}}K\right)$. Among them, the sum term $\sum_{K=K_{min}}^{K_{max}}K=\frac{\left(K_{max}+K_{min}\right)\left(K_{max}-K_{min}+1\right)}{2}$. In this paper, $K_{min}=2$ and $K_{max}=2\sqrt{N}$. Therefore, $\sum_{K=K_{min}}^{K_{max}}K\approx \frac{2\sqrt{N}\cdot 2\sqrt{N}}{2}=2N$. Combined with the characteristic that $I_{K}$ is a constant, the total complexity of the clustering stage can be simplified to: $O\left(I_{K}\cdot N\cdot \sum_{K=K_{min}}^{K_{max}}K\right)=O(N^{2})$.

Note that $K_{min}=2$ is set as a general lower bound to ensure a basic clustered structure and can be flexibly adjusted for different UAV swarm scales.

#### 5.5.2. Computational Complexity of the GPSR Inter-Cluster Route Establishment Stage

GPSR routing is a greedy state routing, and the core operations include neighboring node searching and hop-by-hop iteration. In the worst case, all *N* nodes support all *M* services, and all services are broadcast services. At this time, each service needs to establish at most (*N* − 1) end-to-end routes, and the total number of routes is N·M·(N−1). For each route, the source node needs to select the next hop among (*N* − 1) candidate nodes, and the next-hop node continues to select among the remaining nodes until it reaches the destination node. Theoretically, the total number of possible routes reaches the factorial level. However, the greedy search strategy of GPSR greatly compresses the search space: each node only needs to evaluate the neighboring nodes that are closer to the destination node, and the average number of neighbors is much smaller than *N*. Let the average node degree be *deg*; then the computational complexity of establishing a single route is O(H·deg) Therefore, the complexity of the GPSR stage is O(N·M·N·H·deg). Since *M*, *H*, and *deg* are all constants much smaller than *N*, the complexity of this stage can be simplified to $O(N^{2})$.

In actual engineering scenarios, UAV swarm services are mostly communications between specific nodes, not fully connected broadcasts. Therefore, the actual routing complexity is much lower than the above theoretical maximum value.

#### 5.5.3. Computational Complexity of the Ant Colony Algorithm-Based Power Optimization Stage

The ant colony algorithm only optimizes the power of nodes in the set $\Omega_{K}$, not all nodes in the network, and the power of each node only selects among $m_{i}$ discrete values. Each iteration of the ant colony algorithm needs to calculate the fitness function of each ant, and the calculation of the fitness function needs to traverse all nodes supporting each service. In the worst case, *N* nodes all support *M* services. At this time, the total complexity of the ant colony optimization stage is: $O\left({ANT}_{num}\cdot I_{A}\cdot |\Omega_{K}|\cdot {max(m}_{i})\cdot N\cdot M\right)$. Since ${ANT}_{num}$ and $I_{A}$ are constants, and $\left|\Omega_{K}\right|$, ${max(m}_{i})$, and *M* are much smaller than *N*, the complexity of this stage can be simplified to O(N).

#### 5.5.4. Overall Algorithm Complexity and Theoretical Comparison

The overall computational complexity of the proposed heuristic algorithm is the sum of the complexities of the three stages. According to the above analysis, since the computational complexity of the ant colony algorithm stage is negligible, the overall complexity of the algorithm is dominated by the K-means clustering and GPSR routing stages, namely:

(31) $$O_{total}=O\left(N^{2}\right)+O\left(N^{2}\right)+O\left(N\right)=O\left(N^{2}\right).$$

Compared with the theoretical optimal solution in Section 4.5, the proposed algorithm reduces the super-exponential complexity $O\left({B_{N}\cdot P}^{N}\right)$ to the polynomial complexity $O\left(N^{2}\right)$ through a heuristic strategy.

Compared with the theoretical complexity of the typical clustering algorithm ICW [21], it can be seen that the core complexity of the ICW algorithm comes from the clustering optimization process of the Whale Optimization Algorithm (WOA), and its overall complexity is also $O\left(N^{2}\right)$, which is comparable to the complexity level of the proposed algorithm. Both are polynomial complexities, providing a computationally efficient and fair basis for the performance comparison in the subsequent simulation stage.

## 6. Simulation Analysis

### 6.1. Simulation Scenario

MATLAB R2021a is used to build a UAV swarm simulation scenario to simulate and verify the performance of the proposed algorithm. Nodes move uniformly in a straight line within the range of 10 km × 10 km at a speed of 5 m/s without obstacle avoidance behavior. The basic parameters of the simulation scenario are set as shown in Table 1:

Four types of services are set, and the ratio of packet arrival rates for priority services 0 to 3 is set to 1:2:4:8 [11]. The length of each service data packet is 100 bytes, and the data packet arrival process follows an independent Poisson distribution. The specific parameters of each service are set as shown in Table 2:

The parameter values in the ant colony algorithm are shown in Table 3. These parameters follow typical empirical settings in ant colony optimization studies to ensure stable convergence and balanced search, rather than being tuned for specific performance gains.

Under the above simulation parameter settings, the following first compares the simulation values and theoretical values of the transmission success rate to determine the effectiveness and usability of the quantitative estimation formula. On this basis, the proposed algorithm is simulated and compared with the standalone SPMA protocol and the ICW clustering algorithm.

### 6.2. Simulation Results and Analysis

#### 6.2.1. Simulation Verification of Packet Transmission Success Rate

This section verifies the accuracy of the packet transmission success rate given by Equation (23) through simulation. In a fully connected network, Nodes 1 to 25 broadcast the highest-priority swarm networking service to Node 0 at the same packet arrival rate, and the highest-priority transmission threshold of each node is set sufficiently large so that data is transmitted immediately upon arrival. During this process, the simulated value of the packet transmission success rate is recorded. Meanwhile, the slot transmission probability is obtained by dividing the total number of transmitted packets by the product of the occupancy statistics window length and the number of transmitting nodes, and the theoretical value of the packet transmission success rate is then calculated according to Equations (21)–(23).

**Figure 8:**
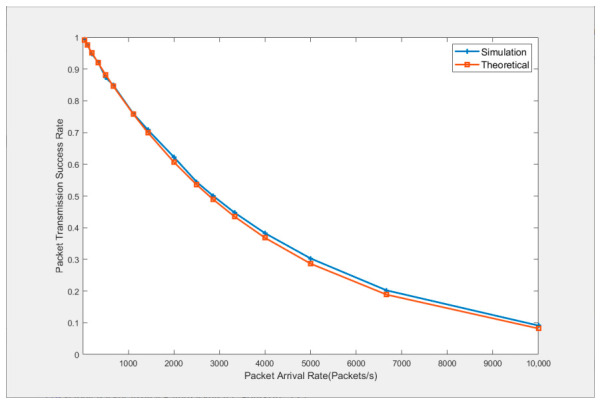
Comparison between simulated and theoretical values of packet transmission success rate.

Figure 8 plots two curves showing the simulation and theoretical values of the packet transmission success rate versus the packet arrival rate at each node. A comparison of the two curves shows that when the packet arrival rate is 40 packets/second, the theoretical transmission success rate is 99.00% and the simulated value is 99.24%, with a relative error well below 1%. When the packet arrival rate reaches 10,000 packets/second, the theoretical value is 8.21% and the simulated value is 9.14%, with a relative error of 10.2%.

To further verify the accuracy of the packet transmission success rate given in Equation (23) when multiple priority services coexist, nodes 1 to 25 are set to send four priority services to node 0 at the same packet arrival rate. According to the theoretical derivation and the simulation results shown in Figure 8, the transmission threshold of priority 0 is set to 12, which corresponds to a packet transmission success rate of no less than 99% for each node when there are 25 nodes and the packet arrival rate is 40 packets/second. In addition, the transmission thresholds of priorities 1 to 3 are set to 10, 8, and 6 respectively.

**Figure 9:**
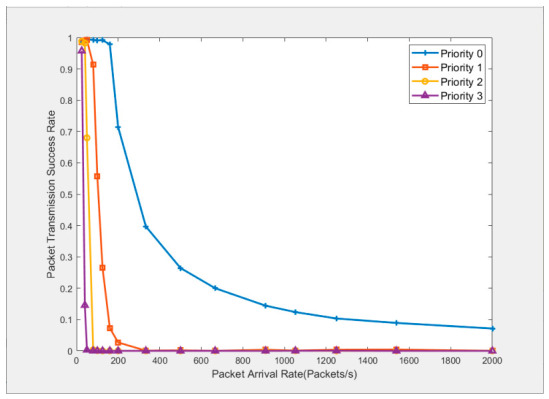
Relationship between packet transmission success rate and arrival rate.

Figure 9 shows the relationship between the packet transmission success rate and the packet arrival rate at each node. It can be seen from the figure that the packet transmission success rate of each priority service gradually decreases with the increase in the packet arrival rate, but the higher the priority, the slower the transmission success rate decreases. When the packet arrival rate exceeds 333 packets/second, the transmission success rates of the other three priority services decrease to nearly zero, but the transmission success rate of priority 0 is still 48.3%.

#### 6.2.2. Algorithm Performance Comparison and Effectiveness Verification

This section analyzes the performance of the proposed algorithm. The transmission thresholds of priorities 0 to 3 are still set to 12, 10, 8, and 6. Priority services 0 and 1 run on all 100 nodes and are broadcast within each cluster and among all cluster heads; priority services 2 and 3 are initiated on 10 randomly selected nodes in the entire network, and 10 destination nodes are also randomly selected in the entire network. When each node sends or forwards a data packet, if it is found that the delay of the data packet has exceeded the specified delay requirement, the packet is immediately discarded. Therefore, the end-to-end delay of successfully transmitted data packets must meet the corresponding delay requirement. When calculating the utility value $U_{K}$, the priority weights $\alpha_{0}$~$\alpha_{3}$ are set to 0.4, 0.3, 0.2, and 0.1 respectively.

**Figure 10:**
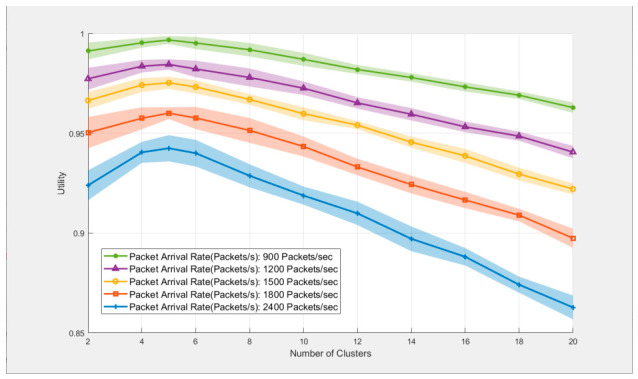
Curve of utility value varying with the number of clusters.

Figure 10 shows the curve of the utility value of the proposed algorithm varying with the number of clusters under five packet arrival rates. Each data point is averaged over 10 independent runs with 95% confidence intervals. It can be seen that the utility value is the maximum when the number of clusters is 5, and then gradually decreases as the number of clusters increases. All metrics within the confidence interval at 5 clusters are higher than those at other cluster numbers, verifying the stability of the optimal cluster number. For example, when the packet arrival rate is 900 packets/second, the mean utility value reaches 99.67% when the number of clusters is 5, and the packet transmission rates of priorities 0 to 3 are 98.73%, 96.24%, 94.34%, and 91.39%, respectively; when the number of clusters increases to 20, the utility value decreases to 96.29%, and the transmission success rates decrease to 97.27%, 92.02%, 89.78%, and 86.64%, respectively.

**Figure 11:**
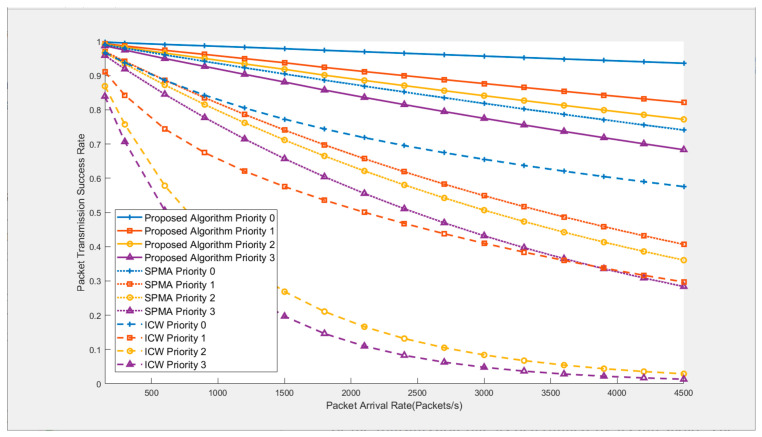
Comparison of packet transmission success rates among different algorithms.

Figure 11 compares the packet transmission success rates of the proposed algorithm (with 5 clusters) with the standalone SPMA protocol and the ICW clustering algorithm. Under the standalone SPMA protocol, to achieve a fully connected network, the transmission power of each node in the simulation is set to exactly cover the farthest node from it. For the ICW algorithm, since it does not specify a specific MAC layer protocol, the SPMA protocol is also used for its MAC layer in the simulation for unified comparison. It can be seen from the figure that the proposed algorithm achieves the best performance, followed by the standalone SPMA algorithm, and the ICW algorithm has the worst performance. Moreover, as the data packet arrival rate increases, the performance advantage of the proposed algorithm becomes more obvious. For example, when the packet arrival rate is 600 packets/second, the transmission success rate of priority 0 for the proposed algorithm is 99.1%, 96.1% for the SPMA protocol, and 88.6% for the ICW algorithm. In terms of the utility value, the proposed algorithm is 7.5% higher than the SPMA algorithm and 31.9% higher than ICW; when the packet arrival rate increases to 4500 packets/second, the transmission success rate of priority 0 is 93.6% for the proposed algorithm, 74.1% for the SPMA protocol, and 57.6% for the ICW algorithm. In terms of the utility value, the proposed algorithm is 63.5% higher than SPMA and 162.3% higher than ICW.

**Figure 12:**
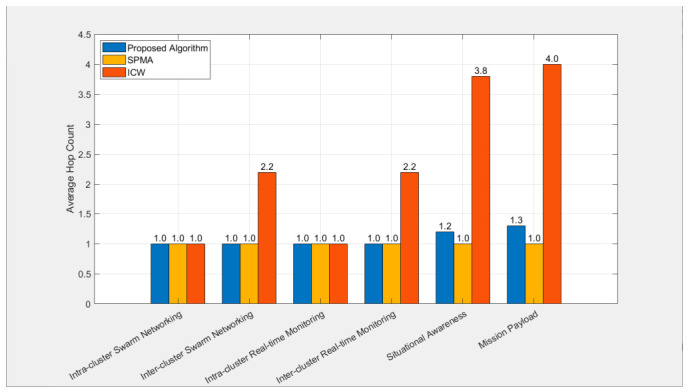
Comparison of average hop counts of different services among three algorithms.

Figure 12 shows the average number of hops of various services under the three algorithms. It can be seen from the figure that under the SPMA protocol, all services are 1 hop; under the proposed algorithm, the intra-cluster and inter-cluster swarm networking and real-time monitoring services are all 1 hop, the average number of hops for inter-cluster situational awareness services is 1.2, and the average number of hops for inter-cluster mission payload services is 1.3; under the ICW algorithm, the intra-cluster swarm networking and real-time monitoring services are all 1 hop, while the average number of hops for inter-cluster swarm networking services and real-time monitoring services is 2.2, the average number of hops for inter-cluster situational awareness services is 3.8, and the average number of hops for inter-cluster mission payload services is 4.0.

The above simulation results are consistent with the design principles of each algorithm: the standalone SPMA algorithm assumes that all nodes are directly connected. The ICW algorithm stipulates that communication between all nodes must be forwarded by cluster head nodes and at the same time stipulates that the transmission power of cluster head nodes will not be increased after clustering is completed. This leads to inter-cluster services often requiring multiple forwards. During the forwarding process, many data packets are actively discarded due to timeout or cannot be correctly received due to collisions on the channel, resulting in a significant decrease in the end-to-end transmission success rate. This indicates that the ICW algorithm cannot meet services with strict delay or transmission success rate requirements. In the proposed algorithm, to reduce the number of forwards, any node can act as a forwarding node, and through the iteration of the ant colony algorithm, a larger transmission power can be used to further reduce the number of forwards for inter-cluster services.

**Figure 13:**
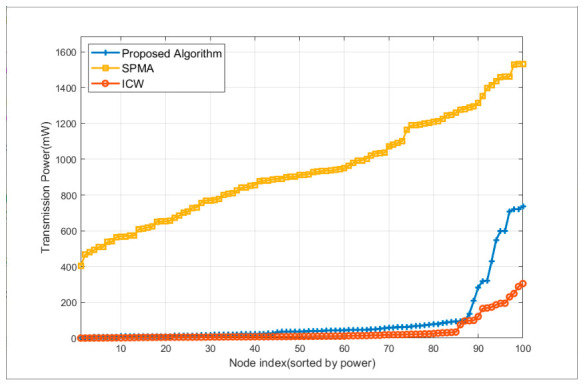
Comparison of node transmission power distribution among three algorithms (nodes sorted by power in ascending order).

Figure 13 shows the transmission power of all 100 nodes under the three algorithms in ascending order, and Table 4 statistically compares the transmission power of each algorithm. It can be seen from the figure that the transmission power of the SPMA protocol is much higher than that of the other two algorithms. The reason for the slow rise in its power curve is the node position: the power of the central node is the smallest, and the power increases as it is farther from the center. The power curves of both the proposed algorithm and the ICW algorithm are obviously divided into two stages: the first stage rises slowly because intra-cluster nodes only need to be directly connected to the cluster head node of their own cluster in one hop. Therefore, the farther a node is away from the cluster head of their own cluster, the greater the power. However, it can be seen from the table that the average value of other nodes in the proposed algorithm is 36.3 mW, which is much larger than 10.6 mW of member nodes in ICW. This is because the number of clusters in the proposed algorithm is 5, while the number of clusters in the ICW algorithm is 17, so the average coverage range of clusters in the proposed algorithm is much larger than that in the ICW algorithm. The latter stage of both algorithms rises rapidly because the nodes undertaking inter-cluster communication need to communicate with other clusters, so the communication distance is farther. However, the proposed algorithm rises more sharply and has higher power because the proposed algorithm allows inter-cluster communication nodes to further increase the transmission power to reduce the number of forwarding hops, but the cluster head nodes of the ICW algorithm do not increase the transmission power after clustering is completed. In terms of overall average value, the average power of all nodes is 89.6 mW for the proposed algorithm, 937.7 mW for the SPMA protocol, and 35.5 mW for the ICW algorithm. The average power of the proposed algorithm is 90.4% lower than that of the SPMA protocol and 152.3% higher than that of the ICW algorithm.

## 7. Discussion

### 7.1. Comparison with Existing SPMA Optimization Methods

Recent research on the SPMA protocol mainly targets fully connected networks and improves performance to a certain extent by optimizing key parameters but cannot alleviate the severe channel contention caused by insufficient frequency resources [7,8,9,10,11,12]. In contrast, the proposed framework reduces interference through spatial partitioning and adaptive power control, which fundamentally compensates for performance degradation caused by the lack of frequency diversity. As verified by simulations, the proposed method reduces transmission power by 90.4% compared with the standard SPMA protocol, while significantly improving the end-to-end transmission success rate under heavy traffic loads.

Nevertheless, DL-based SPMA optimization methods provide meaningful insights for future improvement. For example, BiLSTM-based channel load prediction [10] can better capture fast topology changes in high-mobility UAV networks, and DQN-based access and backoff optimization [12] can process high-dimensional state information. Integrating these DL-based modules into the proposed framework will further strengthen its adaptability to dynamic environments.

### 7.2. Comparison with Existing Clustering Algorithms

A comprehensive comparison between the proposed algorithm and representative clustering algorithms is summarized in Table 5.

The proposed method performs virtual iterative computation before actual deployment, using the derived closed-form transmission success rate expression to quickly converge to a suboptimal solution without real-time message exchange or status measurement. This significantly reduces clustering delay and signaling overhead. Unlike traditional algorithms that rely entirely on cluster heads to forward inter-cluster traffic, the GPSR-based routing mechanism allows any node to serve as a relay, thus reducing path length, transmission delay, and congestion at cluster heads.

Although DL-based clustering algorithms [24,25,26] offer stronger adaptability to highly dynamic topologies, they suffer from high computational complexity, weak interpretability, and large training costs, making them difficult to deploy on resource-constrained UAVs. The proposed model-driven method achieves a more practical balance among performance, complexity, and reliability, making it more suitable for frequency-constrained UAV swarm scenarios with strict real-time requirements.

The current framework focuses on QoS guarantees and interference suppression under quasi-static UAV topology. For subsequent extensions, mobility prediction and dynamic re-clustering will be introduced to maintain stable performance under high-speed maneuvering. Additionally, compact DL modules such as DQN [24] will be combined with K-means to achieve fast initial clustering and intelligent cluster head adaptation, further enhancing robustness and scalability.

## 8. Conclusions

To address the performance limitation of the SPMA protocol in large-scale UAV swarm networking under severely constrained frequency resources, this paper proposes a joint clustering and power optimization method. Simulation results demonstrate that under heavy traffic loads, the proposed method improves the end-to-end packet transmission success rate by 63.5% compared with the standalone SPMA protocol and by 162.3% compared with the ICW algorithm, while reducing the average transmission power by 90.4% relative to SPMA. The highest-priority swarm networking service maintains a delivery probability above 93.6% even at high arrival rates, and the end-to-end delay satisfies the requirement of no more than 2 ms for critical control traffic. These results verify that the proposed scheme effectively balances transmission reliability, power consumption, and latency, providing a practical solution for UAV swarms in frequency-constrained scenarios.

In future work, we will extend the proposed framework to distributed clustering to reduce the dependence on global information. Moreover, imperfect state knowledge, dynamic topology, and mobility-adaptive clustering mechanisms will be investigated to improve the practicality in realistic UAV swarm deployments.

## Figures and Tables

**Table 1:** Basic parameters of the simulation scenario.

Parameter	Value	Parameter	Value
Network range	10 km × 10 km	Receive antenna gain	1
Number of nodes	100	System loss coefficient	2
Carrier frequency	2.4 GHz	Receiver sensitivity	−90 dBm
Transmission power	0~5 W	Transmission rate	20 Mbps
Transmit antenna gain	1	Frame length	10 μs

**Table 2:** Service parameter settings.

Priority	Service Type	Delay	Transmission Success Rate	Communication Mode
0	Swarm networking	≤2 ms	≥99%	Broadcast
1	Real-time monitoring	≤10 ms	≥97%	Broadcast
2	Situational awareness	≤50 ms	≥95%	Unicast
3	Mission payload	≤100 ms	≥90%	Unicast

**Table 3:** Ant colony parameter values.

Parameter	Symbol	Value
Number of ants	${ANT}_{num}$	80
Number of iterations	*I_A_*	50
Pheromone evaporation factor	ρ	0.12
Heuristic factor	λ	1.5
Pheromone enhancement coefficient	*Q*	6

**Table 4:** Statistical comparison of node transmission power under three algorithms.

Algorithm	Mean (mW)	Standard Deviation (mW)	Maximum (mW)	Minimum (mW)
Proposed algorithm—Cluster head/forwarding nodes	278.6	253.5	736.4	7.9
Proposed algorithm—Other nodes	36.3	28.3	136.5	0.3
SPMA	937.7	284.6	1533.2	403.9
ICW—Cluster head nodes	176.5	70.7	305.0	75.7
ICW—Cluster member nodes	10.6	8.1	34.2	0.5

**Table 5:** Performance and mechanism comparison of different clustering algorithms.

Comparison Item	Proposed Algorithm	Existing Algorithms
Primary Goal	Maximize multi-service QoS (end-to-end transmission success rate)	Balance energy consumption and communication performance
Iteration Mechanism	Virtual iteration: Converges theoretically before deployment	Real-time iteration: Requires multiple operational rounds to converge
Computational Complexity	Low complexity: Relies on derived mathematical formulas	High complexity; requires real-time feedback of multiple parameters
Inter-Cluster Routing	GPSR-based arbitrary node forwarding, avoids cluster head bottlenecks	Cluster-head-only forwarding, causing bottlenecks
QoS Guarantee	Quantifiable QoS guarantees: Derived performance for given clustering and power	Best-effort service without strict guarantees
Interpretability	Strong; theoretically complete	Weak; black-box

## Data Availability

This study includes all the data used in this work.
